# Efficacy and safety of pharmacological and non-pharmacological therapies in Lennox-Gastaut syndrome: a systematic review and network meta-analysis

**DOI:** 10.3389/fphar.2025.1522543

**Published:** 2025-02-26

**Authors:** Zhengyan Zhu, Zhenpan Zhang, Wei Xiao, Chunhua Wang, Risheng Liang

**Affiliations:** ^1^ Department of Neurosurgery, Fujian Medical University Union Hospital, Fuzhou, China; ^2^ Department of Neurosurgery, Fujian Neurosurgical Institute, Fuzhou, China; ^3^ Department of Neurosurgery, Xiangtan Central Hospital, Xiangtan, China

**Keywords:** Lennox-Gastaut syndrome, seizures, antiepileptic drugs, non-pharmacological therapies, network meta-analysis

## Abstract

**Objective:**

This study aimed to evaluate the efficacy and safety of antiepileptic drugs and non-pharmacological treatments in patients with Lennox-Gastaut syndrome (LGS).

**Methods:**

We conducted a systematic search of the PubMed, Embase, Cochrane, and Web of Science databases for randomized controlled trials (RCTs) evaluating both pharmacological and non-pharmacological interventions for LGS. The treatments assessed included cannabidiol, fenfluramine, clobazam, rufinamide, felbamate, lamotrigine, topiramate, deep brain stimulation, and anterior corpus callosotomy. The primary efficacy outcome was defined as a reduction of at least 50% in the frequency of drop seizures during treatment compared to baseline levels. The secondary efficacy outcome was measured as the median percentage reduction in monthly drop seizure frequency throughout the treatment period. Safety assessments were based on the incidence of adverse events and serious adverse events. All outcomes were ranked according to their surface under the cumulative ranking curve (SUCRA).

**Result:**

This network meta-analysis encompassed 12 RCTs involving a total of 1,445 patients. The SUCRA indicated that clobazam 1 mg/kg/day, anterior corpus callosotomy, and rufinamide were the three most effective interventions for achieving a reduction of at least 50% in drop seizures. In terms of median percentage reduction in drop seizure frequency, clobazam 1 mg/kg/day ranked highest, followed by clobazam 0.5 mg/kg/day and rufinamide. Regarding safety profiles, SUCRA analysis revealed that cannabidiol 20 mg/kg/day had the highest likelihood of inducing adverse events, followed closely by fenfluramine 0.7 mg/kg/day. Lamotrigine was found to be most likely to cause serious adverse reactions, with cannabidiol 10 mg/kg/day following closely behind.

**Conclusion:**

Clobazam 1 mg/kg/day, anterior corpus callosotomy, and rufinamide manifested the most optimal efficacy in seizure control among LGS patients. Caution should be exercised when administering cannabidiol, lamotrigine, and fenfluramine 0.7 mg/kg/day in clinical practice to mitigate safety concerns associated with drug-related side effects.

## Introduction

LGS, a rare but gravely severe form of developmental epileptic encephalopathy (DEE), preponderantly afflicts children, with an approximated incidence of roughly 2 per 100,000 individuals (Heiskala, 1997; Durá-Travé et al., 2007). LGS represents 1%–4% of all childhood epilepsy cases and exhibits a higher prevalence in males compared to females (Durá-Travé et al., 2007; Trevathan et al., 1997) A distinctive feature of LGS is the manifestation of multiple seizure varieties, substantial cognitive impairments and coupled with aberrant electroencephalogram (EEG) manifestations characterized by slow spike-and-wave complexes (Arzimanoglou et al., 2009). The seizure types associated with LGS include tonic seizures, atonic seizures, and atypical absence seizures. Tonic and atonic seizures can result in increased muscle tone or weakness, leading to “drop attacks.” These manifestations can adversely affect the quality of life for both patients and their families. Consequently, enhancing the management strategies for LGS has emerged as a prominent area of research interest.

The treatment strategies for LGS encompass both pharmacological and non-pharmacological approaches. To date, the U.S. Food and Drug Administration has sanctioned six antiseizure medications (ASMs) for LGS: lamotrigine, topiramate, felbamate, rufinamide, clobazam, and clonazepam (Döring et al., 2016). Recently, two randomized controlled trials have demonstrated that cannabidiol can significantly reduce the frequency of drop attacks associated with LGS, thus establishing it as a novel therapeutic option for this condition (Devinsky et al., 2018; Thiele et al., 2018). While these medications can reduce seizure frequency to some degree, they seldom achieve complete remission, and many patients continue to experience refractory seizures. Furthermore, the simultaneous administration of multiple ASMs often leads to adverse effects such as sedation, cognitive dysfunction, and gastrointestinal issues, thereby complicating the management of the condition. Considering the limitations inherent in pharmacological treatments, there is a growing interest in non-pharmacological interventions, including electrical stimulation and surgical options. Electrical stimulation offers a less invasive alternative and has shown considerable promise in refractory cases. Surgical procedures, such as anterior corpus callosotomy, may be contemplated for patients with refractory LGS. Owing to the extensive array of accessible therapeutic modalities, their variable efficacy, and the ongoing debates regarding optimal dosing strategies, this study aims to systematically evaluate and compare the therapeutic effectiveness of both pharmacological and non-pharmacological interventions for LGS via a network meta-analysis. In contradistinction to recent network meta-analyses, this research will concurrently appraise different dosages of pharmacological treatments in conjunction with non-pharmacological alternatives, thereby furnishing novel perspectives on therapeutic choices for patients afflicted with LGS.

## Methods

### Search strategy

This study was performed and reported in compliance with the Preferred Reporting Items for Systematic Reviews and Meta-Analyses (PRISMA) extension statement for network meta-analyses (Sterne et al., 2019). A systematic search was conducted across the Cochrane, PubMed, Embase, and Web of Science databases to identify randomized controlled trials (RCTs), with a search cutoff date of 1 August 2024. The search strategy utilized a combination as follows: ((Lennox Gastaut Syndrome [MeSH Terms]) OR ((Lennox Gastaut Syndromes [Title/Abstract]) OR (Lennox-Gastaut Syndrome [Title/Abstract]))) AND (randomized controlled trial [Publication Type] OR randomized [Title/Abstract] OR placebo [Title/Abstract]), with no restrictions on language.

### Eligibility criteria

Initially, articles were deemed eligible if they met specific criteria (Heiskala, 1997): patients diagnosed with LGS, regardless of sex, race, or geographic location (Durá-Travé et al., 2007); the intervention group receiving various therapeutic interventions while the control group received usual treatment (sham surgery or placebo) (Trevathan et al., 1997); studies included must be RCTs (Arzimanoglou et al., 2009); studies must provide detailed information on the efficacy and adverse events associated with the therapeutic interventions. Exclusion criteria comprised duplicate publications, animal studies, case reports, conference abstracts, reviews, unavailable full texts, and studies involving other organic diseases.

### Outcome measures

The primary efficacy outcome was defined as a reduction of at least 50% in the frequency of drop seizures during treatment compared to baseline levels. The secondary efficacy outcome was the median percentage reduction in the frequency of drop seizures over the treatment period. Drop seizures are defined as epileptic seizures (atonic, tonic, or tonic–clonic) involving the entire body, trunk, or head that lead or could lead to a fall, injury, or slumping in a chair. Safety outcomes were evaluated based on the occurrence of treatment-related adverse events and serious adverse events.

### Data extraction and assessment of the risk of bias

Two authors conducted a comprehensive literature review based on predefined inclusion and exclusion criteria. In cases of disagreement, discrepancies were resolved through discussion or by consulting a third party to achieve consensus. The extracted information from the included studies encompassed essential details such as the first author, year of publication, country of origin, sample size, gender distribution, age range, intervention measures, and outcome metrics. The Cochrane Collaboration’s tool was used to assess the quality of included trials (Jansen et al., 2008), by which each study was classified as high, low, or unclear risk of bias in accordance with the following criteria: random sequence generation, allocation concealment, blinding of outcome participants and personnel, blinding of outcome assessment, incomplete outcome data, selective reporting, and other biases. The quality of eligible studies was assessed by two investigators independently, and disagreements were resolved by discussion and coordinated by the third investigator.

### Statistical analysis

We conducted a Bayesian network meta-analysis utilizing R version 4.3.2 software (R Foundation for Statistical Computing) with *a priori* fuzzy random effects models applied to multiple sets of trials. The combined estimates and probabilities of each treatment being the most effective were derived using Markov chain Monte Carlo methods. Model convergence was evaluated through trajectory plots and Brooks-Gelman-Rubin plots, while dichotomous classification results were expressed as posterior odds ratios along with their corresponding 95% confidence intervals (CIs). We calculated the surface under the cumulative ranking curve (SUCRA) to estimate the probability of optimal intervention. Network diagrams were generated using STATA version 15.0 with an appropriate pass-through macro command loaded. In these diagrams, each circle represents a drug, and edges indicate existing comparisons; the size of each circle is proportional to the number of patients included in the analysis. Cumulative probability plots were created using the ggplot2 package.

## Results

### Literature search

**FIGURE 1:**
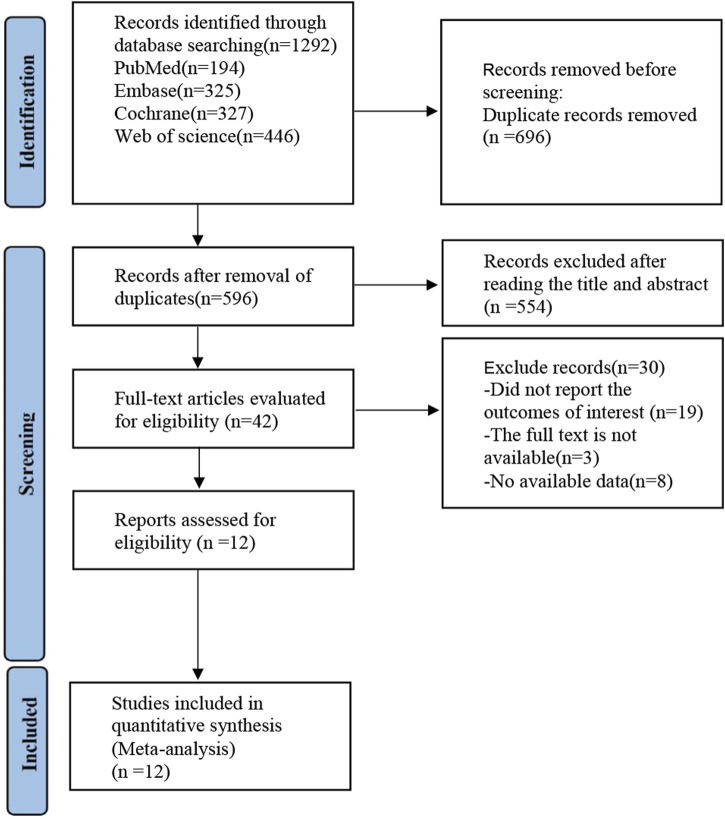
Study flow diagram.

A preliminary search of the databases identified 1,292 articles, of which 696 duplicates were removed. Among the remaining articles, 554 were excluded due to their classification as letters, editorials, reviews, conference abstracts, or because the study design (observational or retrospective studies) did not meet the inclusion criteria. Following a thorough review of the full texts, 30 studies were excluded for failing to report the outcomes of interest or lacking detailed results. Ultimately, 12 articles (Devinsky et al., 2018; Thiele et al., 2018; Dalic et al., 2022; Efficacy of felbamate in childhood, 1993; Glauser et al., 2008; Knupp et al., 2022; Liang et al., 2014; McMurray and Striano, 2016; Motte et al., 1997; Ng et al., 2011; Ohtsuka et al., 2014; Sachdeo et al., 1999) were included in the analysis (Figure 1).

### Characteristics of the included studies and risk of bias assessment

**FIGURE 2:**
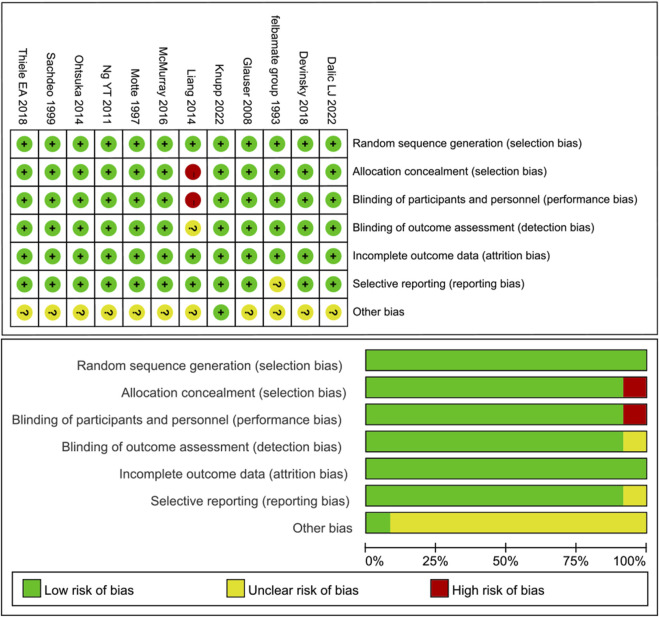
Risk of bias.

A total of 12 RCTs were included, encompassing 1,445 patients diagnosed with Lennox-Gastaut syndrome (LGS). Among these studies, 235 patients received cannabidiol across two RCTs, while 123 patients were treated with rufinamide in three RCTs. The remaining seven RCTs involved patients receiving deep brain stimulation (DBS) (n = 10), anterior corpus callosotomy (n = 23), fenfluramine (n = 176), felbamate (n = 37), lamotrigine (n = 79), clobazam (n = 179), and topiramate (n = 48). In total, 633 patients were randomly assigned to the control group, which received usual treatments across all trials. For studies in which drug dosages were not explicitly reported, the maximum dose within the specified treatment range was utilized. Detailed characteristics of the included studies are summarized in Table 1. Among the 12 RCTs, one was rated as having a high risk of bias, while the remaining eleven were assessed as having a low risk of bias. All studies clearly described the blinding methods employed; the high risk was primarily attributed to deviations from intended interventions. The assessment of bias risk for the included studies is illustrated in Figure 2.

**TABLE 1:** Characteristics of the study participants.

Study	Year	Country	Sample size	Gender (M/F)	Mean age	Intervention	Outcomes
Glauser	2008	United States	Rufinamide:74 UT:64	86/52	Rufinamide:13 UT:10.5	Rufinamide:45 mg/kg/d	F1,F2; F3; F4
Dalic	2022	Australia	DBS:10 UT:9	6/13	DBS:24.4 UT:25	Deep brain stimulation	F1; F2; F3; F4
Liang	2014	China	Callosotomy:23 UT:37	38/22	Callosotomy:9.48 UT:9.73	Anterior corpus callosotomy	F1; F4
Devinsky	2018	United States	Cannabidiol:149 UT:76	129/96	Cannabidiol:15.7 UT:15.3	Cannabidiol:10 mg/kg/d Cannabidiol:20 mg/kg/d	F1; F2; F3; F4
Knupp	2022	United States	Fenfluramine:176 UT:87	146/117	Fenfluramine:13 UT:14	Fenfluramine:0.2 mg/kg/d Fenfluramine:0.7 mg/kg/d	F1; F2; F3; F4
Felbamate group	1993	United States	Felbamate:37 UT:36	51/22	Felbamate:12 UT:14	Felbamate:45 mg/kg/d	F2; F3
Motte	1997	United States	Lamotrigine:79 UT:90	99/70	Lamotrigine:9.6 UT:10.9	Lamotrigine:18 mg/kg/d	F2; F3; F4
Ohtsuka	2016	Japan	Rufinamide:28 UT:30	33/21	Rufinamide:16 UT:13.9	Rufinamide:45 mg/kg/d	F1; F2; F3; F4
Thiele	2018	United States	Cannabidiol:86 UT:85	88/83	Cannabidiol:15.4 UT:15.2	Cannabidiol:20 mg/kg/d	F1; F2; F3; F4
Ng YT	2011	United States	Clobazam:179 UT:59	144/94	Clobazam:12.2 UT:13	Clobazam:0.25 mg/kg/d Clobazam:0.5 mg/kg/d Clobazam:1 mg/kg/d	F1; F2; F3; F4
Sachdeo	1999	United States	Topiramate:48 UT:50	53/45	Topiramate:11.2 UT:11.2	Topiramate:6 mg/kg/d	F2; F3; F4
McMurray	2016	United Kingdom	Rufinamide:21 UT:10	20/11	Rufinamide:25.2 UT:29.3	Rufinamide:45 mg/kg/d	F1; F2; F4

UT, Usual treatment (sham-operated group or placebo group); DBS, deep brain stimulation; F1, adverse events; F2, Median Percent Reductions in Monthly Drop-Seizure Frequency during the Treatment Period; F3, serious adverse events; F4,Reductions of at Least 50% from Baseline in Drop-Seizure Frequency during the Treatment Period.

### Efficacy outcomes

#### Primary outcome

**FIGURE 3:**
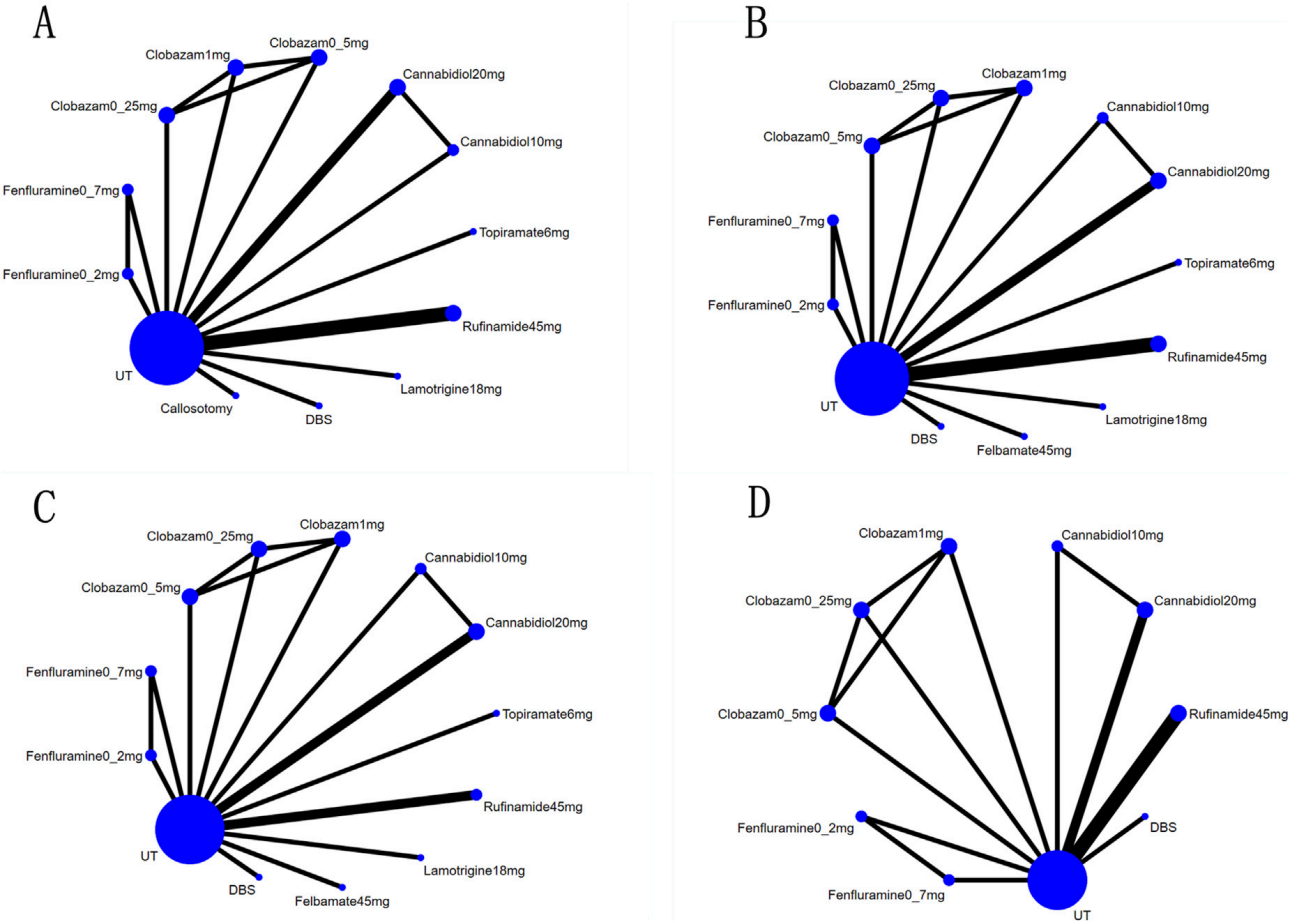
Network map for all outcomes: **(A)**, Network map for reductions of at Least 50% from Baseline in Drop-Seizure Frequency during the Treatment Period; **(B)**, Network map for median Percent Reductions in Monthly Drop-Seizure Frequency during the Treatment Period; **(C)**, Network map for serious adverse events; **(D)**, Network map for adverse events.

**FIGURE 4:**
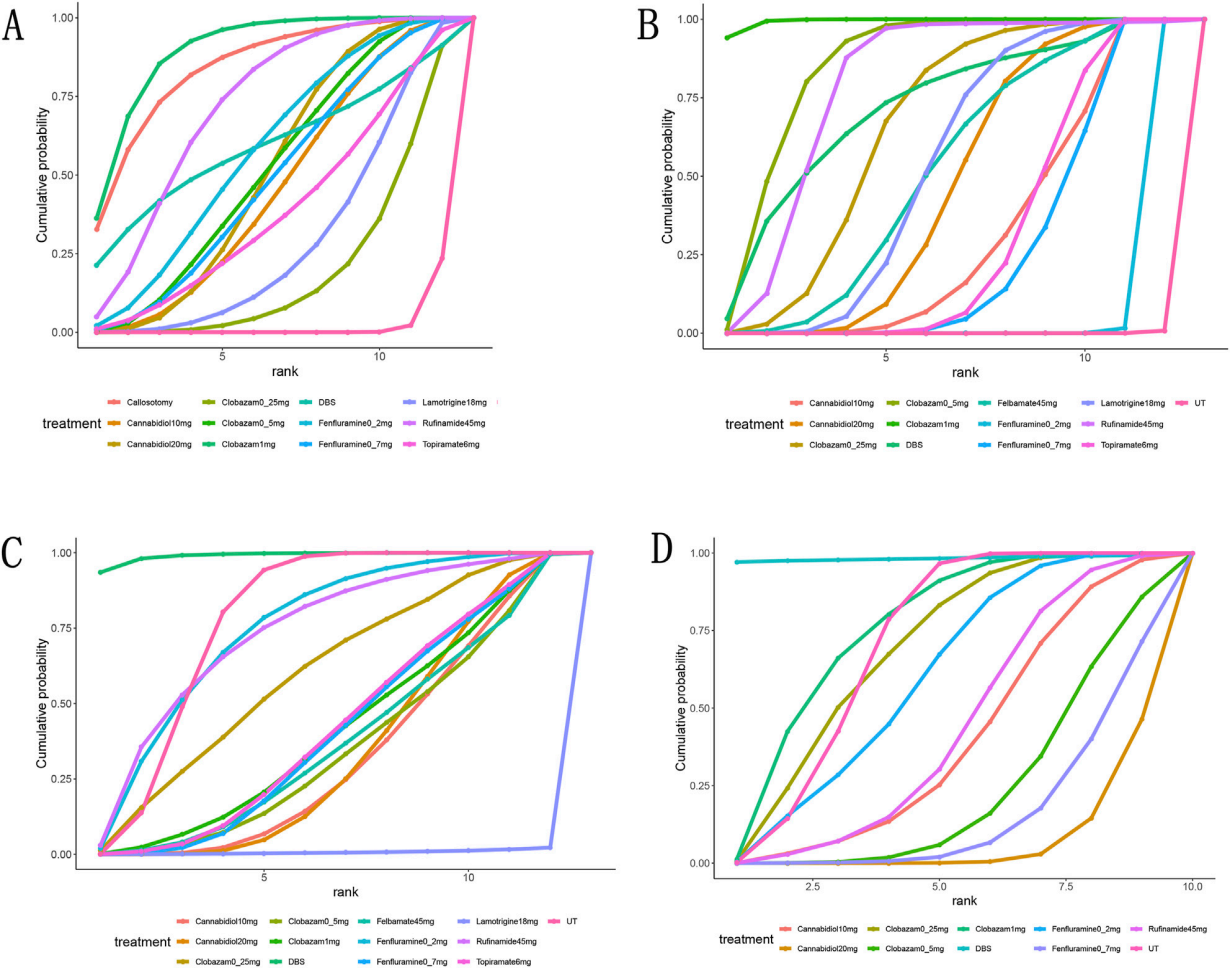
Surface under the cumulative ranking curve probabilities for the ranking. **(A)**, at Least 50% from baseline in drop-seizure frequency during the treatment period; **(B)**, median percent reductions in monthly drop-seizure frequency during the treatment period; **(C)**, serious adverse events; **(D)**, adverse events.

**FIGURE 5:**
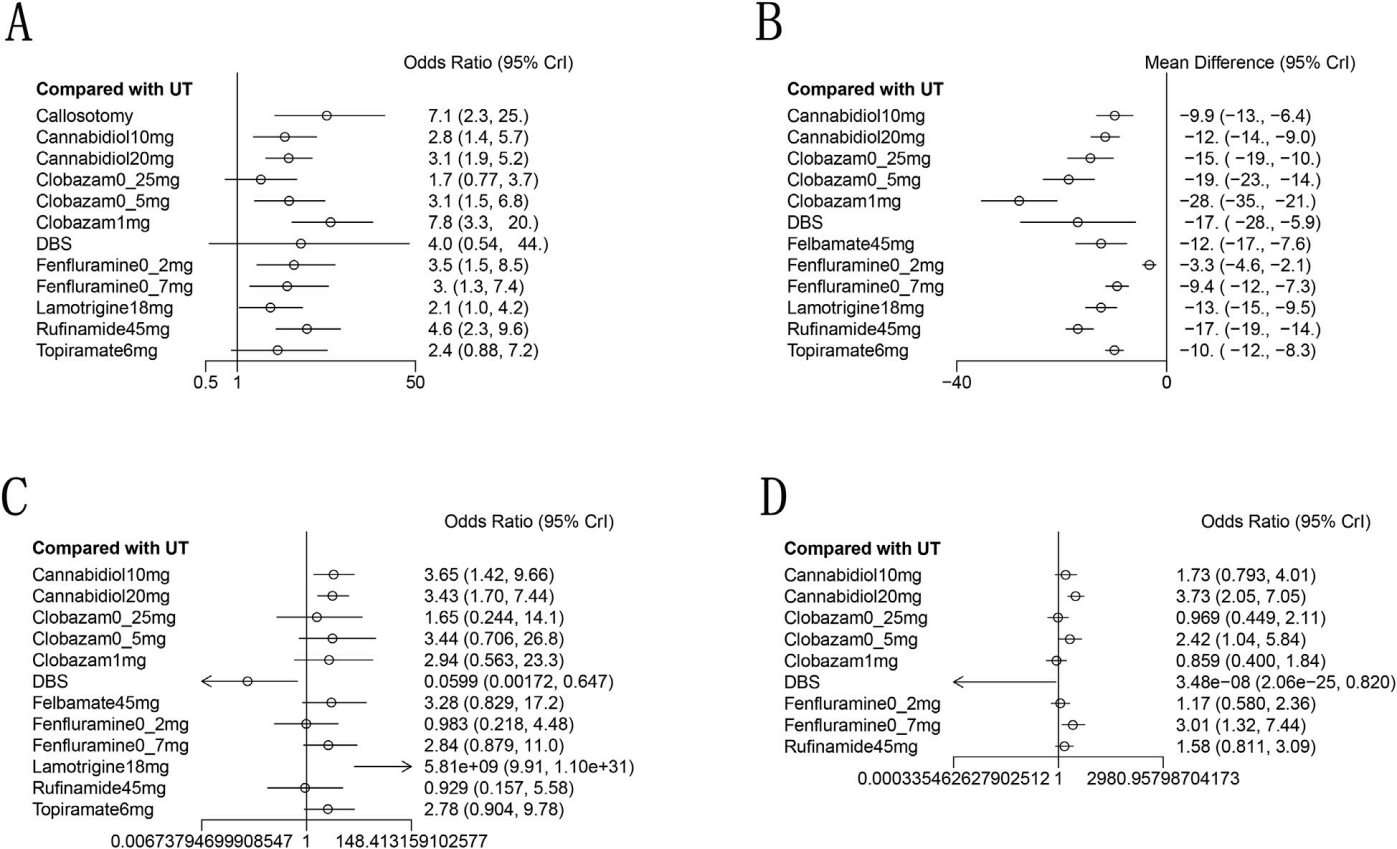
Forest plot for the efficacy and safety outcomes: **(A)**, at Least 50% from baseline in drop-seizure frequency during the treatment period; **(B)**, median percent reductions in monthly drop-seizure frequency during the treatment period; **(C)**, serious adverse events; **(D)**, adverse events.

A total of 11 studies (Devinsky et al., 2018; Thiele et al., 2018; Dalic et al., 2022; Glauser et al., 2008; Knupp et al., 2022; Liang et al., 2014; McMurray and Striano, 2016; Motte et al., 1997; Ng et al., 2011; Ohtsuka et al., 2014; Sachdeo et al., 1999) reported a reduction of at least 50% in the frequency of drop seizures during treatment compared to baseline. The network graph for efficacy comparisons is presented in Figure 3A. SUCRA suggested that clobazam 1 mg/kg/day (89.7%), anterior corpus callosotomy (84.3%), and rufinamide 45 mg/kg/day (72.1%) emerged as the top three treatments in terms of ranking probability (Figure 4A; Table 2). Compared to usual treatment, anterior corpus callosotomy [OR = 7.1, 95% CI (2.3, 25)], cannabidiol 10 mg/kg/day [OR = 2.8, 95% CI (1.4, 5.7)], cannabidiol 20 mg/kg/day [OR = 3.1, 95% CI (1.9, 5.2)], clobazam 0.5 mg/kg/day [OR = 3.1, 95% CI (1.5, 6.8)], clobazam 1 mg/kg/day [OR = 7.8, 95% CI (3.3, 20)], fenfluramine 0.2 mg/kg/day [OR = 3.5, 95% CI (1.5, 8.5)], fenfluramine 0.7 mg/kg/day [OR = 3, 95% CI (1.3, 7.4)], and rufinamide 45 mg/kg/day [OR = 4.6, 95% CI (2.3, 9.6)] significantly reduced the incidence of drop seizures in patients with LGS (Figure 5A). Furthermore, in relation to this outcome, low-dose clobazam 0.25 mg/kg/day [OR = 0.22, 95% CI (0.09, 0.5)] and moderate-dose clobazam 0.5 mg/kg/day [OR = 0.4, 95% CI (0.16, 0.93)] were determined to be less effective than high-dose clobazam 1 mg/kg/day (Table 3).

**TABLE 2:** Ranking according to SUCRA for efficacy and safety outcomes Lower.

Treatment	F1 (%)	F2 (%)	F3 (%)	F4 (%)
Cannabidiol:10 mg/kg/d	39.1	31.5	32.8	45.6
Cannabidiol:20 mg/kg/d	7.1	47	34.5	50.9
Clobazam:0.25 mg/kg/d	68.6	66.0	60.0	19.8
Clobazam:0.5 mg/kg/d	23.1	85.0	35.4	51.5
Clobazam:1 mg/kg/d	75.3	99.4	41.0	89.7
DBS	98.3	71.9	99.1	59.3
Fenfluramine:0.2 mg/kg/d	59.7	8.5	74.8	57.7
Fenfluramine:0.7 mg/kg/d	15.4	26.5	40.7	48.7
Rufinamide:45 mg/kg/d	43.0	78.2	73.4	72.1
Anterior corpus callosotomy	NA	NA	NA	84.3
Felbamate:45 mg/kg/d	NA	51.8	37.3	NA
Lamotrigine:18 mg/kg/d	NA	53.0	0.7	29.2
Topiramate:6 mg/kg/d	NA	30.6	42.1	39.0
UT	70.3	0.076	78	2.1

SUCRA values correspond to higher probabilities of worse safety (F1,F3), and higher SUCRA values correspond to higher probabilities of better efficacy (F2,F4).

**TABLE 3:** Network analysis of efficacy.

F1: OR 95%CI												
Cannabidiol10mg												
0.46 (0.19, 1.14)	Cannabidiol20mg											
1.77 (0.59, 5.45)	3.84 (1.44, 10.32)*	Clobazam0_25mg										
0.71 (0.22, 2.3)	1.54 (0.53, 4.41)	0.4 (0.17, 0.93)	Clobazam0_5mg									
2.01 (0.67, 6.13)	4.35 (1.65, 11.67)*	1.13 (0.53, 2.43)	2.81 (1.23, 6.76)*	Clobazam1mg								
53054215.79 (1.9, 1.24643653043255e+25)*	113903619.71 (4.19, 2.64751995632895e+25)*	29855928.2 (1.08, 6.90652356063824e+24)*	73458459.28 (2.7, 1.75200741804761e+25)*	26436248.79 (0.95, 6.1163583862229e+24)	DBS							
1.48 (0.52, 4.35)	3.2 (1.27, 8.2)*	0.84 (0.29, 2.35)	2.07 (0.7, 6.27)	0.74 (0.26, 2.05)	0 (0, 0.77)*	Fenfluramine0_2mg						
0.57 (0.17, 1.86)	1.25 (0.42, 3.5)	0.32 (0.1, 1.01)	0.81 (0.24, 2.67)	0.29 (0.09, 0.88)	0 (0, 0.3)*	0.39 (0.16, 0.9)*	Fenfluramine0_7mg					
1.09 (0.39, 3.16)	2.37 (0.97, 5.89)	0.62 (0.22, 1.7)	1.54 (0.53, 4.6)	0.54 (0.2, 1.48)	0 (0, 0.56)*	0.74 (0.28, 1.93)	1.9 (0.66, 5.78)	Rufinamide45mg				
1.72 (0.79, 3.94)	3.74 (2.06, 7.04)*	0.98 (0.45, 2.1)	2.43 (1.05, 5.82)*	0.86 (0.4, 1.84)	0 (0, 0.87)*	1.17 (0.58, 2.37)	2.99 (1.32, 7.38)*	1.58 (0.82, 3.09)	UT			

F2: MD 95% CI												
Cannabidiol10mg												
1.8 (-2.29, 5.96)	Cannabidiol20mg											
4.62 (-0.94, 10.14)	2.81 (-2.29, 7.93)	Clobazam0_25mg										
8.75 (2.81, 14.69)*	6.94 (1.39, 12.5)*	4.11 (-2.18, 10.44)	Clobazam0_5mg									
18.2 (10.21, 26.21)*	16.39 (8.73, 24.04)*	13.58 (5.4, 21.78)*	9.45 (0.95, 17.94)*	Clobazam1mg								
7.05 (-4.44, 18.49)	5.22 (-6.08, 16.45)	2.39 (-9.39, 14.19)	-1.74 (-13.67, 10.19)	-11.18 (-24.26, 1.89)	DBS							
2.59 (-3.39, 8.55)	0.79 (-4.79, 6.35)	-2.03 (-8.55, 4.41)	-6.16 (-13.03, 0.68)	-15.61 (-24.31, -6.98)*	-4.46 (-16.41, 7.55)	Felbamate45mg						
-6.6 (-10.25, -2.92)*	-8.41 (-11.36, -5.45)*	-11.23 (-15.73, -6.71)*	-15.34 (-20.35, -10.32)*	-24.8 (-32.09, -17.48)*	-13.64 (-24.61, -2.59)*	-9.18 (-14.19, -4.15)*	Fenfluramine0_2mg					
-0.45 (-4.52, 3.63)	-2.26 (-5.7, 1.19)	-5.08 (-9.88, -0.23)*	-9.2 (-14.49, -3.88)*	-18.64 (-26.14, -11.14)*	-7.48 (-18.58, 3.7)	-3.04 (-8.34, 2.32)	6.15 (3.8, 8.49)*	Fenfluramine0_7mg				
2.62 (-1.93, 7.13)	0.81 (-3.19, 4.78)	-2.01 (-7.25, 3.2)	-6.13 (-11.84, -0.43)*	-15.59 (-23.38, -7.84)*	-4.41 (-15.72, 6.91)	0.03 (-5.63, 5.69)	9.22 (5.98, 12.38)*	3.07 (-0.59, 6.7)	Lamotrigine18mg			
6.99 (2.2, 11.18)*	5.19 (1.03, 8.72)*	2.34 (-3.19, 7.23)	-1.77 (-7.83, 3.67)	-11.25 (-19.63, -3.66)*	-0.1 (-12.18, 11.16)	4.39 (-1.7, 9.8)	13.61 (10.41, 16.18)*	7.46 (3.7, 10.58)*	4.37 (-0.02, 8.18)	Rufinamide45mg		
0.05 (-3.76, 3.89)	-1.76 (-4.9, 1.39)	-4.58 (-9.22, 0.08)	-8.7 (-13.82, -3.56)*	-18.15 (-25.53, -10.78)*	-6.99 (-17.99, 4.09)	-2.54 (-7.65, 2.59)	6.65 (4.54, 8.75)*	0.49 (-2.23, 3.22)	-2.56 (-5.97, 0.84)	-6.96 (-9.78, -3.51)*	Topiramate6mg	
-9.9 (-13.35, -6.43)*	-11.71 (-14.39, -9.02)*	-14.52 (-18.85, -10.18)*	-18.64 (-23.5, -13.78)*	-28.09 (-35.28, -20.91)*	-16.93 (-27.83, -5.95)*	-12.5 (-17.3, -7.6)*	-3.3 (-4.55, -2.05)*	-9.45 (-11.59, -7.3)*	-12.52 (-15.43, -9.53)*	-16.92 (-19.16, -13.99)*	-9.95 (-11.61, -8.26)*	UT

F3: OR 95%CI												
Cannabidiol10mg												
1.06 (0.47, 2.34)	Cannabidiol20mg											
2.21 (0.22, 19.47)	2.1 (0.22, 16.76)	Clobazam0_25mg										
1.05 (0.11, 6.76)	1 (0.12, 5.8)	0.48 (0.09, 1.99)	Clobazam0_5mg									
1.24 (0.13, 8.38)	1.18 (0.13, 7.26)	0.56 (0.1, 2.52)	1.17 (0.33, 4.41)	Clobazam1mg								
60.37 (4.62, 2151.06)*	56.93 (4.78, 1951.22)*	28.26 (1.25, 1576.9)*	59.96 (3.13, 3195.91)*	51.19 (2.61, 2681.81)*	DBS							
1.1 (0.17, 5.96)	1.04 (0.17, 5.05)	0.49 (0.04, 6.37)	1.04 (0.11, 12.08)	0.89 (0.09, 10.48)	0.02 (0, 0.29)*	Felbamate45mg						
3.76 (0.63, 22.55)	3.56 (0.67, 19.52)	1.7 (0.15, 22.83)	3.6 (0.4, 43.85)	3.06 (0.32, 37.72)	0.06 (0, 1.04)	3.44 (0.44, 31.42)	Fenfluramine0_2mg					
1.28 (0.25, 5.82)	1.21 (0.26, 4.93)	0.57 (0.05, 6.43)	1.21 (0.15, 12.41)	1.03 (0.12, 10.82)	0.02 (0, 0.3)*	1.16 (0.17, 8.73)	0.34 (0.09, 1.1)	Fenfluramine0_7mg				
0 (0, 0.38)*	0 (0, 0.36)*	0 (0, 0.2)*	0 (0, 0.41)*	0 (0, 0.36)*	0 (0, 0.01)*	0 (0, 0.37)*	0 (0, 0.11)*	0 (0, 0.3)*	Lamotrigine18mg			
3.92 (0.51, 29.07)	3.71 (0.53, 25.4)	1.76 (0.13, 27.62)	3.75 (0.34, 53.88)	3.19 (0.27, 46.8)	0.06 (0, 1.27)	3.59 (0.36, 38.92)	1.03 (0.1, 10.69)	3.08 (0.36, 28.3)	5686802661.03 (9.35, 1.57734613767195e+31)*	Rufinamide45mg		
1.3 (0.27, 5.74)	1.23 (0.29, 4.78)	0.58 (0.06, 6.52)	1.23 (0.16, 12.59)	1.05 (0.13, 10.91)	0.02 (0, 0.31)*	1.19 (0.18, 8.66)	0.34 (0.05, 2.29)	1.02 (0.18, 5.98)	1881784518.59 (3.37, 4.9354571449587e+30)*	0.33 (0.04, 2.8)	Topiramate6mg	
3.65 (1.43, 9.54)*	3.44 (1.7, 7.39)*	1.64 (0.24, 13.91)	3.42 (0.71, 26.49)	2.92 (0.56, 22.95)	0.06 (0, 0.65)*	3.29 (0.83, 17.22)	0.97 (0.21, 4.41)	2.83 (0.89, 11.13)	5328621584.77 (10.07, 1.43808954714402e+31)*	0.93 (0.16, 5.66)	2.8 (0.91, 9.82)	UT

F4: OR 95% CI												
Callosotomy												
2.52 (0.65, 10.63)	Cannabidiol10mg											
2.31 (0.66, 9.09)	0.92 (0.48, 1.73)	Cannabidiol20mg										
4.27 (1.06, 18.8)*	1.69 (0.59, 4.8)	1.85 (0.72, 4.7)	Clobazam0_25mg									
2.29 (0.57, 9.98)	0.91 (0.32, 2.55)	0.99 (0.39, 2.48)	0.54 (0.25, 1.14)	Clobazam0_5mg								
0.92 (0.21, 4.21)	0.36 (0.12, 1.09)	0.4 (0.14, 1.07)	0.22 (0.09, 0.5)*	0.4 (0.16, 0.93)*	Clobazam1mg							
1.76 (0.13, 18.92)	0.7 (0.06, 5.95)	0.76 (0.07, 6.11)	0.41 (0.03, 3.62)	0.77 (0.06, 6.75)	1.93 (0.15, 17.63)	DBS						
2.05 (0.48, 9.31)	0.81 (0.26, 2.38)	0.89 (0.32, 2.33)	0.48 (0.15, 1.5)	0.9 (0.28, 2.77)	2.23 (0.65, 7.6)	1.16 (0.13, 14.08)	Fenfluramine0_2mg					
2.37 (0.55, 10.79)	0.94 (0.3, 2.78)	1.03 (0.37, 2.73)	0.56 (0.17, 1.76)	1.04 (0.32, 3.26)	2.59 (0.75, 8.89)	1.34 (0.15, 16.48)	1.16 (0.59, 2.28)	Fenfluramine0_7mg				
3.44 (0.89, 14.46)	1.36 (0.52, 3.63)	1.49 (0.63, 3.5)	0.81 (0.28, 2.28)	1.5 (0.53, 4.24)	3.76 (1.24, 11.75)*	1.95 (0.23, 23.05)	1.68 (0.57, 5.21)	1.45 (0.49, 4.49)	Lamotrigine18mg			
1.54 (0.4, 6.52)	0.61 (0.23, 1.62)	0.67 (0.28, 1.56)	0.36 (0.13, 1.03)	0.67 (0.24, 1.91)	1.68 (0.55, 5.27)	0.87 (0.1, 10.31)	0.75 (0.25, 2.31)	0.65 (0.22, 2)	0.45 (0.17, 1.19)	Rufinamide45mg		
2.93 (0.61, 14.7)	1.17 (0.32, 3.99)	1.27 (0.39, 3.97)	0.69 (0.18, 2.5)	1.28 (0.34, 4.65)	3.2 (0.81, 12.66)	1.67 (0.17, 22.02)	1.44 (0.37, 5.51)	1.24 (0.32, 4.77)	0.85 (0.24, 2.94)	1.9 (0.53, 6.68)	Topiramate6mg	
7.11 (2.28, 25.45)*	2.83 (1.43, 5.69)*	3.09 (1.87, 5.17)*	1.68 (0.77, 3.68)	3.12 (1.46, 6.83)*	7.78 (3.33, 19.54)*	4.04 (0.54, 43.54)	3.47 (1.54, 8.54)*	3 (1.32, 7.39)*	2.07 (1.05, 4.18)*	4.61 (2.35, 9.61)*	2.43 (0.88, 7.2)	UT

Network meta-analysis results of the efficacy in terms of odd ratio (OR) or mean deviation (MD) for responder rate, which are reported in order of surface under the curve cumulative ranking. Top-ranked treatment listed in the top left corner and rankings proceed down the diagonal. A value with an asterisk indicates a statistically significant result. A, at Least 50% from baseline in drop-seizure frequency during the treatment period; B, median percent reductions in monthly drop-seizure frequency during the treatment period; C, serious adverse events; D, adverse events.

#### Secondary outcome

A total of 11 studies (Devinsky et al., 2018; Thiele et al., 2018; Dalic et al., 2022; Efficacy of felbamate in childhood, 1993; Glauser et al., 2008; Knupp et al., 2022; McMurray and Striano, 2016; Motte et al., 1997; Ng et al., 2011; Ohtsuka et al., 2014; Sachdeo et al., 1999) reported the median percentage reduction in the frequency of drop seizures. The network plot comparing efficacy is presented in Figure 3B. The SUCRA indicated that the three most effective treatments were clobazam 1 mg/kg/day (99.4%), clobazam 0.5 mg/kg/day (85%), and rufinamide 45 mg/kg/day (78.2%) (Figure 4B; Table 2). Compared to usual treatment, cannabidiol 10 mg/kg/day [MD = −9.9, 95% CI (−13, −6.4)], cannabidiol 20 mg/kg/day [MD = −12, 95% CI (−14, −9.0)], clobazam 0.25 mg/kg/day [MD = −15, 95% CI (−19, −10)], clobazam 0.5 mg/kg/day [MD = −19, 95% CI (−23, −14)], clobazam 1 mg/kg/day [MD = −28, 95% CI (−35, −21)], DBS [MD = −17, 95% CI (−28, −5.9)], felbamate 45 mg/kg/day [MD = −12, 95% CI (−17, −7.6)], fenfluramine 0.2 mg/kg/day [MD = −3.3, 95% CI (−4.6, −2.1)], fenfluramine 0.7 mg/kg/day [MD = −9.4, 95% CI (−12, −7.3)], lamotrigine 18 mg/kg/day [MD = −13, 95% CI (−15, −9.5)], rufinamide 45 mg/kg/day [MD = −17, 95% CI (−19, −14)], and topiramate 6 mg/kg/day [MD = −10, 95% CI (−12, 8.3)] all significantly reduced the median frequency of drop seizures (Figure 5B). In this outcome measure, cannabidiol 20 mg/kg/day was less effective than clobazam at 1 mg/kg/day [MD = 16.39, 95% CI (8.73, 24.04)] and rufinamide 45 mg/kg/day [MD = 5.19, 95% CI (1.03, 8.72)]. Clobazam 1 mg/kg/day outperformed lamotrigine 18 mg/kg/day [MD = −15.59, 95% CI (−23.38, −7.84)] and rufinamide at 45 mg/kg/day [MD = −11.25, 95% CI (−19.63, −3.66)]. Fenfluramine 0.2 mg/kg/day was less effective than fenfluramine at 0.7 mg/kg/day [MD = 6.15, 95% CI (3.8, 8.49)] (Table 3).

### Safety outcomes

Eight randomized controlled trials (Thiele et al., 2018; Sterne et al., 2019; Efficacy of felbamate in childhood, 1993; Knupp et al., 2022; Liang et al., 2014; Motte et al., 1997; Ohtsuka et al., 2014; Sachdeo et al., 1999) provided comprehensive data regarding adverse events. The most frequently reported adverse events included drowsiness, fever, gastrointestinal disturbances, and cognitive impairment. According to the SUCRA (Figure 4D), DBS (98.3%), clobazam 1 mg/kg/day (75.3%), and clobazam 0.25 mg/kg/day (68.6%) exhibited a lower risk of adverse reactions. In contrast, patients receiving cannabidiol 20 mg/kg/day (7.1%) had the highest incidence of adverse events, followed by those treated with fenfluramine 0.7 mg/kg/day (15.4%). Compared to usual treatment, the likelihood of experiencing adverse events was significantly higher for cannabidiol 20 mg/kg/day [OR = 3.73, 95% CI (2.05, 7.05)], clobazam 0.5 mg/kg/day [OR = 2.42, 95% CI (1.04, 5.84)], and fenfluramine 0.7 mg/kg/day [OR = 3.01, 95% CI (1.32, 7.44)] (Figure 5D). Additionally, cannabidiol 20 mg/kg/day [OR = 4.35, 95% CI (1.65, 11.67)] was associated with a higher risk of adverse reactions compared to clobazam 1 mg/kg/day. Notably, the risk of adverse events with fenfluramine 0.2 mg/kg/day [OR = 0.39, 95% CI (0.16, 0.90)] was lower than that observed with fenfluramine 0.7 mg/kg/day (Table 3).

In addition to reporting the incidence of adverse events, ten studies (Devinsky et al., 2018; Thiele et al., 2018; Dalic et al., 2022; Efficacy of felbamate in childhood, 1993; Glauser et al., 2008; Knupp et al., 2022; Motte et al., 1997; Ng et al., 2011; Ohtsuka et al., 2014; Sachdeo et al., 1999) provided comprehensive information regarding serious adverse events (SAEs). SAEs were defined as events that resulted in hospitalization, permanent discontinuation of treatment, or posed life-threatening risks. According to the SUCRA, lamotrigine 18 mg/kg/day showed the highest probability of SAEs (0.7%), followed by cannabidiol 10 mg/kg/day (32.8%) and cannabidiol 20 mg/kg/day (34.5%) (Figure 4C). In contrast, DBS (99.1%), fenfluramine 0.2 mg/kg/day (74.8%), and rufinamide at 45 mg/kg/day (73.4%) were associated with a substantially lower risk of SAEs. Compared to usual treatment, the odds of experiencing SAEs were significantly elevated with cannabidiol 10 mg/kg/day [OR = 3.65, 95% CI (1.42, 9.66)], cannabidiol 20 mg/kg/day [OR = 3.43, 95% CI (1.70, 7.44)], and lamotrigine 18 mg/kg/day [OR = 5.81e + 09, 95% CI (9.91, 1.10e + 31)] (Figure 5C).

### Publication bias

**FIGURE 6:**
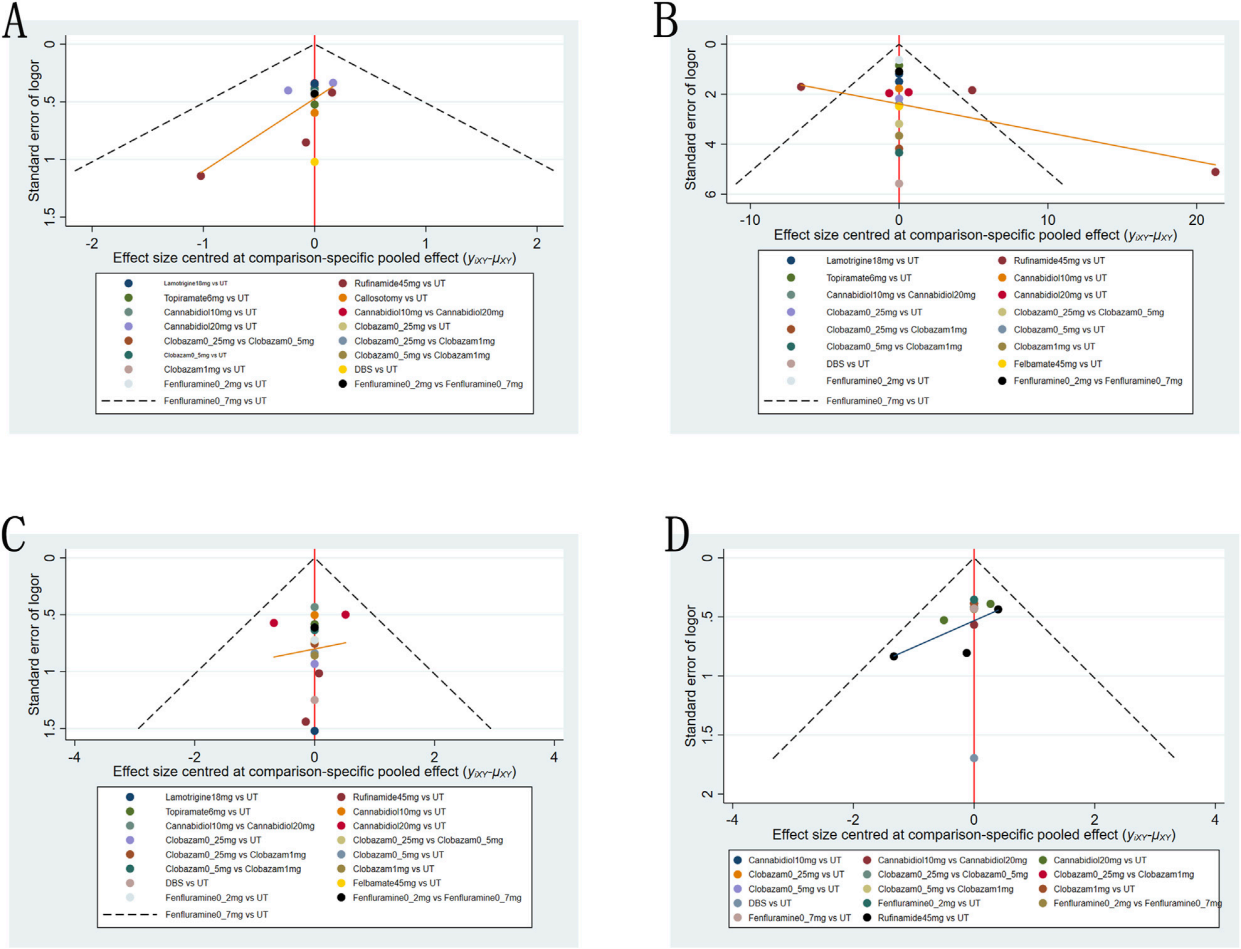
Funnel plot for the efficacy and safety outcomes: **(A)**, at Least 50% from baseline in drop-seizure frequency during the treatment period; **(B)**, median percent reductions in monthly drop-seizure frequency during the treatment period; **(C)**, serious adverse events; **(D)**, adverse events.

We employed funnel plots to assess publication bias across four outcomes: a reduction in drop-seizure frequency of at least 50% from baseline; the median percentage reduction in drop-seizure frequency; adverse reactions and serious adverse reactions. The results suggest a high possibility of publication bias for all four outcomes (Figure 6).

## Discussion

To the best of our knowledge, this is the first study to systematically evaluate the effects of various dosages and types of antiepileptic drugs, along with non-pharmacological treatments, on LGS. A prior analysis indicated that antiepileptic medications significantly reduce seizure incidence compared to placebo (Zhang L. et al., 2022); however, it did not investigate the influence of drug dosages. This network meta-analysis further quantifies the effects of different drug dosages and provides a more precise treatment ranking. Moreover, for the first time, this study compares surgical treatment methods (DBS and anterior corpus callosotomy) with antiepileptic drugs in LGS patients, thereby elucidating the relative efficacy and safety profiles of various treatment regimens. These findings offer valuable insights for clinicians in making informed treatment decisions.

We evaluated the efficacy and safety of cannabidiol, fenfluramine, clobazam, rufinamide, felbamate, lamotrigine, topiramate, DBS, and anterior corpus callosotomy in the treatment of LGS through the inclusion of twelve randomized controlled trials. The results indicated that, with respect to achieving a minimum of 50% reduction in the frequency of drop seizures compared to baseline, clobazam administered at a dosage of 1 mg/kg/d exhibited a significant advantage, followed by anterior corpus callosotomy and rufinamide at 45 mg/kg/d. In terms of median percentage reduction in drop seizure frequency as an outcome measure, clobazam at 1 mg/kg/d demonstrated superior efficacy, succeeded by clobazam at 0.5 mg/kg/d,with rufinamide at 45 mg/kg/d ranking third. These medications exert their anticonvulsant effects through distinct mechanisms of action. Our study suggests that high-dose clobazam is more effective than its lower-dose counterpart. Clobazam is a 1,5-benzodiazepine that exerts its effects by binding to GABA_A_ receptors, thereby enhancing the frequency of chloride channel opening. This mechanism results in an increased influx of chloride ions, leading to neuronal hyperpolarization. Consequently, clobazam mitigates excessive excitability within the central nervous system, thereby reducing seizure frequency (Gauthier and Mattson, 2015). Rufinamide primarily attenuates excessive neuronal excitation and abnormal discharges by delaying the activity of voltage-dependent sodium channels. This mechanism inhibits the generation and propagation of action potentials, thereby restoring overexcited neurons to baseline levels (Lin et al., 2022). Furthermore, recent studies have demonstrated that rufinamide can also modulate the activity of large-conductance Ca2^+^-activated K^+^ channels, enhancing potassium ion influx and consequently reducing neuronal excitability (Lai et al., 2022). Cannabidiol has demonstrated anti-epileptic effects in various animal models (Rosenberg et al., 2017; Do Val-da Silva et al., 2017; Klein et al., 2017). Due to its effectiveness in decreasing seizure frequency during Phase 3 clinical trials, the U.S. Food and Drug Administration granted approval for cannabidiol as a therapeutic option for seizures associated with Dravet Syndrome and Lennox-Gastaut Syndrome (LGS) in 2018 (US Food and Drug Administration, 2018). Cannabidiol enhances the strength of the M current mediated by the Kv2/7.3 channel, thereby reducing neuronal excitability and contributing to its anti-epileptic effects (Zhang HB. et al., 2022). Additionally, cannabidiol can inhibit G protein-coupled receptor GPR55 or block transient receptor potential vanilloid subtype (TRPV) ion channels, which in turn inhibits the activity of voltage-dependent L-type calcium channels and further reduces neuronal excitability (Del and Barker-Haliski, 2023; Ibeas Bih et al., 2015). Neuroinflammation significantly contributes to the development of human epilepsy. Cannabidiol exerts its anti-inflammatory effects through the downregulation of the NF-κB pathway and modulation of the IFN-β/STAT signaling cascade (Martinez et al., 2023). Anterior corpus callosotomy is recommended for individuals with drug-resistant epilepsy, especially those experiencing severe drop attacks, such as those associated with Lennox-Gastaut syndrome. This surgical procedure involves severing the anterior fibers of the corpus callosum to prevent the propagation of abnormal electrical signals between the two hemispheres of the brain, thereby reducing both the frequency and severity of seizures. Given that the anterior fibers are less associated with cognitive functions, this intervention typically does not result in significant adverse effects on a patient’s intelligence or cognitive abilities (Maehara and Shimizu, 2001).

Furthermore, safety assessments reveal that the frequency of adverse reactions associated with high-dose cannabidiol (20 mg/kg/day) is greater than that observed with usual treatment. The most frequently reported adverse events related to cannabidiol include drowsiness, decreased appetite, and diarrhea. In many patients receiving cannabidiol treatment concurrently with valproate, liver aminotransferase levels can rise to three times or more above the normal upper limit, thereby heightening the risk of hepatotoxicity and often requiring cessation of treatment (Miller et al., 2020). During the course of treatment, adverse events associated with fenfluramine therapy included decreased appetite and weight loss. These effects may be attributed to the release of serotonin (5-HT) induced by fenfluramine, which stimulates 5-HT2C receptors in pro-opiomelanocortin (POMC) neurons within the hypothalamic melanocortin system that regulates energy homeostasis and feeding behavior (He et al., 2021). Fenfluramine was initially utilized for the treatment of obesity based on its mechanism of action. However, in 1997, it was globally withdrawn from the market following reports that high doses of fenfluramine (60–120 mg/d) were associated with an increased risk of valvular heart disease (Cardiac valvulopathy associated with exposure, 1997). Recent studies have demonstrated that during epilepsy management, no cases of valvular heart disease or pulmonary arterial hypertension were observed following echocardiographic monitoring (Lai et al., 2020; Agarwal et al., 2022). This suggests that the potential benefits of fenfluramine for patients with epilepsy may outweigh its cardiovascular risks. However, this does not imply that fenfluramine is entirely safe for the cardiovascular system. Therefore, when administering high-dose fenfluramine, it remains imperative to closely monitor patients’ cardiac function to ensure safety. Although adverse events associated with fenfluramine treatment can be easily triggered, serious adverse events are infrequently observed. Some preclinical studies have demonstrated that fenfluramine may enhance the survival rate of epileptic mice by mitigating respiratory arrest, neuroinflammation, demyelination, and cell apoptosis induced by seizures (Tupal and Faingold, 2021). While additional research is required to validate these mechanisms, the majority of clinical trials demonstrate favorable tolerability of oral fenfluramine in both short-term and long-term contexts (Knupp et al., 2023; Lagae et al., 2019; Nabbout et al., 2020). Lamotrigine may cause serious skin adverse reactions, such as Stevens-Johnson syndrome (SJS). SJS can be triggered by the formation of toxic metabolites via the minor metabolic pathway of lamotrigine and the depletion of glutathione and L-carnitine (Vázquez et al., 2018). Valproate inhibits the glucuronidation of lamotrigine, thereby reducing its clearance (Kavitha et al., 2015). Consequently, long-term co-administration of lamotrigine and valproic acid may increase the risk of serious adverse reactions. Therefore, when lamotrigine is combined with valproic acid, the dose should be strictly monitored to prevent potential serious adverse reactions.

In patients with LGS, antiepileptic drugs have shown significant efficacy in reducing drop seizures. In contrast to prior research, this study provides the first thorough comparison of non-pharmacological interventions and different dosages of antiepileptic drugs, thereby clarifying the relative efficacy and safety profiles of various treatment regimens. However, this study does possess certain limitations. First, the limited sample size in some randomized controlled trials may compromise the stability of the results. While both anterior corpus callosotomy and DBS have demonstrated promising outcomes in the treatment of LGS, the limited sample size restricts a thorough evaluation of their clinical efficacy and applicability. Second, due to insufficient detailed data on adverse events reported by certain studies, we are unable to fully evaluate the safety of all treatment options. Since the randomized controlled trials included in this review compared various treatment modalities with a conventional treatment group, differences in effect sizes among different modalities were derived from indirect comparisons. Variability in effect sizes across trials and treatment modalities may influence their ranking. Consequently, caution should be exercised regarding these rankings, underscoring the need for future head-to-head studies. Additionally, this study did not incorporate long-term assessments of patients’ quality of life following treatment and changes in neurocognitive function. Future research should address these outcomes to comprehensively evaluate the overall impact of the treatment. Early RCTs of drugs such as felbamate, clobazam, and topiramate were conducted several years ago, while more recent RCTs have evaluated newer agents like cannabidiol and fenfluramine. This considerable time gap may lead to changes in the drug-resistant characteristics of LGS across studies. Consequently, the heterogeneity between patient populations in older versus more recent clinical trials could impact the final analysis of treatment efficacy.

## Conclusion

In this network meta-analysis, we conducted a comprehensive evaluation of the effects of various treatment options on the frequency of drop seizures and the incidence of safety events in patients with LGS. Clobazam, anterior corpus callosotomy, and rufinamide exhibited superior efficacy and safety in reducing fall seizures among LGS patients, particularly high-dose clobazam (1 mg/kg/day), which ranked highly for both efficacy and safety. However, some medications demonstrated significant efficacy but were associated with a high risk of adverse events, indicating that clinical application should be approached with caution and dosages adjusted appropriately (e.g., cannabidiol, fenfluramine, and lamotrigine). These findings provide essential evidence-based guidance for the clinical management of LGS and further support the optimization of individualized treatment regimens.

## Data Availability

The original contributions presented in the study are included in the article/Supplementary Material, further inquiries can be directed to the corresponding author.

## References

[B3] Agarwal A. Farfel G. M. Gammaitoni A. R. Wong P. C. Pinto F. J. Galer B. S. (2022). Long-term cardiovascular safety of fenfluramine in patients with Dravet syndrome treated for up to 3 years: findings from serial echocardiographic assessments. Eur. J. Paediatr. Neurol. 39, 35–39. 10.1016/j.ejpn.2022.05.006 35640431

[B4] Arzimanoglou A. French J. Blume W. T. Cross J. H. Ernst J. P. Feucht M. (2009). Lennox-Gastaut syndrome: a consensus approach on diagnosis, assessment, management, and trial methodology. Lancet Neurol. 8 (1), 82–93. 10.1016/S1474-4422(08)70292-8 19081517

[B2] Cardiac valvulopathy associated with exposure (1997). Cardiac valvulopathy associated with exposure to fenfluramine or dexfenfluramine: U.S. Department of Health and Human Services interim public health recommendations, November 1997. MMWR Morb. Mortal. Wkly. Rep. 46 (45), 1061–1066. 9385873

[B5] Dalic L. J. Warren A. E. L. Bulluss K. J. Thevathasan W. Roten A. Churilov L. (2022). DBS of thalamic centromedian nucleus for lennox-gastaut syndrome (ESTEL trial). Ann. Neurol. 91 (2), 253–267. 10.1002/ana.26280 34877694

[B6] Del P. A. Barker-Haliski M. (2023). Cannabidiol reveals a disruptive strategy for 21st century epilepsy drug discovery. Exp. Neurol. 360, 114288. 10.1016/j.expneurol.2022.114288 36471511 PMC9789191

[B7] Devinsky O. Patel A. D. Cross J. H. Villanueva V. Wirrell E. C. Privitera M. (2018). Effect of cannabidiol on drop seizures in the lennox-gastaut syndrome. N. Engl. J. Med. 378 (20), 1888–1897. 10.1056/NEJMoa1714631 29768152

[B8] Döring J. H. Lampert A. Hoffmann G. F. Ries M. (2016). Thirty years of orphan drug legislation and the development of drugs to treat rare seizure conditions: a cross sectional analysis. PLoS One 11 (8), e0161660. 10.1371/journal.pone.0161660 27557111 PMC4996488

[B9] Do Val-da Silva R. A. Peixoto-Santos J. E. Kandratavicius L. De Ross J. B. Esteves I. De Martinis B. S. (2017). Protective effects of cannabidiol against seizures and neuronal death in a rat model of mesial temporal lobe epilepsy. Front. Pharmacol. 8, 131. 10.3389/fphar.2017.00131 28367124 PMC5355474

[B10] Durá-Travé T. Yoldi-Petri M. E. Gallinas-Victoriano F. (2007). Epilepsy in children in Navarre, Spain: epileptic seizure types and epileptic syndromes. J. Child. Neurol. 22 (7), 823–828. 10.1177/0883073807304207 17715273

[B1] Efficacy of felbamate in childhood (1993). Efficacy of felbamate in childhood epileptic encephalopathy (Lennox-Gastaut syndrome). N. Engl. J. Med. 328 (1), 29–33. 10.1056/NEJM199301073280105 8347179

[B11] Gauthier A. C. Mattson R. H. (2015). Clobazam: a safe, efficacious, and newly rediscovered therapeutic for epilepsy. CNS Neurosci. Ther. 21 (7), 543–548. 10.1111/cns.12399 25917225 PMC6495194

[B12] Glauser T. Kluger G. Sachdeo R. Krauss G. Perdomo C. Arroyo S. (2008). Rufinamide for generalized seizures associated with Lennox-Gastaut syndrome. Neurology 70 (21), 1950–1958. 10.1212/01.wnl.0000303813.95800.0d 18401024

[B13] He Y. Cai X. Liu H. Conde K. M. Xu P. Li Y. (2021). 5-HT recruits distinct neurocircuits to inhibit hunger-driven and non-hunger-driven feeding. Mol. Psychiatry 26 (12), 7211–7224. 10.1038/s41380-021-01220-z 34290371 PMC8776930

[B14] Heiskala H. (1997). Community-based study of Lennox-Gastaut syndrome. Epilepsia 38 (5), 526–531. 10.1111/j.1528-1157.1997.tb01136.x 9184597

[B15] Ibeas Bih C. Chen T. Nunn A. V. Bazelot M. Dallas M. Whalley B. J. (2015). Molecular targets of cannabidiol in neurological disorders. Neurotherapeutics 12 (4), 699–730. 10.1007/s13311-015-0377-3 26264914 PMC4604182

[B16] Jansen J. P. Crawford B. Bergman G. Stam W. (2008). Bayesian meta-analysis of multiple treatment comparisons: an introduction to mixed treatment comparisons. Value Health 11 (5), 956–964. 10.1111/j.1524-4733.2008.00347.x 18489499

[B17] Kavitha S. Anbuchelvan T. Mahalakshmi V. Sathya R. Sabarinath T. R. Gururaj N. (2015). Stevens-Johnson syndrome induced by a combination of lamotrigine and valproic acid. J. Pharm. Bioallied Sci. 7 (Suppl. 2), S756–S758. 10.4103/0975-7406.163545 26538961 PMC4606703

[B18] Klein B. D. Jacobson C. A. Metcalf C. S. Smith M. D. Wilcox K. S. Hampson A. J. (2017). Evaluation of cannabidiol in animal seizure models by the epilepsy therapy screening program (ETSP). Neurochem. Res. 42 (7), 1939–1948. 10.1007/s11064-017-2287-8 28478594

[B19] Knupp K. G. Scheffer I. E. Ceulemans B. Sullivan J. Nickels K. C. Lagae L. (2023). Fenfluramine provides clinically meaningful reduction in frequency of drop seizures in patients with Lennox-Gastaut syndrome: interim analysis of an open-label extension study. Epilepsia 64 (1), 139–151. 10.1111/epi.17431 36196777 PMC10099582

[B20] Knupp K. G. Scheffer I. E. Ceulemans B. Sullivan J. E. Nickels K. C. Lagae L. (2022). Efficacy and safety of fenfluramine for the treatment of seizures associated with lennox-gastaut syndrome: a randomized clinical trial. JAMA Neurol. 79 (6), 554–564. 10.1001/jamaneurol.2022.0829 35499850 PMC9062770

[B21] Lagae L. Sullivan J. Knupp K. Laux L. Polster T. Nikanorova M. (2019). Fenfluramine hydrochloride for the treatment of seizures in Dravet syndrome: a randomised, double-blind, placebo-controlled trial. Lancet 394 (10216), 2243–2254. 10.1016/S0140-6736(19)32500-0 31862249

[B22] Lai M. C. Wu S. N. Huang C. W. (2022). Rufinamide, a triazole-derived antiepileptic drug, stimulates Ca(2+)-activated K(+) currents while inhibiting voltage-gated Na(+) currents. Int. J. Mol. Sci. 23 (22), 13677. 10.3390/ijms232213677 36430153 PMC9697614

[B23] Lai W. W. Galer B. S. Wong P. C. Farfel G. Pringsheim M. Keane M. G. (2020). Cardiovascular safety of fenfluramine in the treatment of Dravet syndrome: analysis of an ongoing long-term open-label safety extension study. Epilepsia 61 (11), 2386–2395. 10.1111/epi.16638 32809271 PMC7754414

[B24] Liang S. Zhang S. Hu X. Zhang Z. Fu X. Jiang H. (2014). Anterior corpus callosotomy in school-aged children with Lennox-Gastaut syndrome: a prospective study. Eur. J. Paediatr. Neurol. 18 (6), 670–676. 10.1016/j.ejpn.2014.05.004 24912732

[B25] Lin Y. C. Lai Y. C. Lin T. H. Yang Y. C. Kuo C. C. (2022). Selective stabilization of the intermediate inactivated Na(+) channel by the new-generation anticonvulsant rufinamide. Biochem. Pharmacol. 197, 114928. 10.1016/j.bcp.2022.114928 35063442

[B26] Maehara T. Shimizu H. (2001). Surgical outcome of corpus callosotomy in patients with drop attacks. Epilepsia 42 (1), 67–71. 10.1046/j.1528-1157.2001.081422.x 11207787

[B27] Martinez N. N. Kelly J. Corna G. Golino M. Abbate A. Toldo S. (2023). Molecular and cellular mechanisms of action of cannabidiol. Molecules 28 (16), 5980. 10.3390/molecules28165980 37630232 PMC10458707

[B28] McMurray R. Striano P. (2016). Treatment of adults with lennox-gastaut syndrome: further analysis of efficacy and safety/tolerability of rufinamide. Neurol. Ther. 5 (1), 35–43. 10.1007/s40120-016-0041-9 26861566 PMC4919131

[B29] Miller I. Scheffer I. E. Gunning B. Sanchez-Carpintero R. Gil-Nagel A. Perry M. S. (2020). Dose-ranging effect of adjunctive oral cannabidiol vs placebo on convulsive seizure frequency in Dravet syndrome: a randomized clinical trial. JAMA Neurol. 77 (5), 613–621. 10.1001/jamaneurol.2020.0073 32119035 PMC7052786

[B30] Motte J. Trevathan E. Arvidsson J. F. Barrera M. N. Mullens E. L. Manasco P. (1997). Lamotrigine for generalized seizures associated with the lennox-gastaut syndrome. Lamictal lennox-gastaut study group. N. Engl. J. Med. 337 (25), 1807–1812. 10.1056/NEJM199712183372504 9400037

[B31] Nabbout R. Mistry A. Zuberi S. Villeneuve N. Gil-Nagel A. Sanchez-Carpintero R. (2020). Fenfluramine for treatment-resistant seizures in patients with Dravet syndrome receiving stiripentol-inclusive regimens: a randomized clinical trial. JAMA Neurol. 77 (3), 300–308. 10.1001/jamaneurol.2019.4113 31790543 PMC6902175

[B32] Ng Y. T. Conry J. A. Drummond R. Stolle J. Weinberg M. A. OV-1012 Study Investigators (2011). Randomized, phase III study results of clobazam in Lennox-Gastaut syndrome. Neurology 77 (15), 1473–1481. 10.1212/WNL.0b013e318232de76 21956725 PMC12477877

[B33] Ohtsuka Y. Yoshinaga H. Shirasaka Y. Takayama R. Takano H. Iyoda K. (2014). Rufinamide as an adjunctive therapy for Lennox-Gastaut syndrome: a randomized double-blind placebo-controlled trial in Japan. Epilepsy Res. 108 (9), 1627–1636. 10.1016/j.eplepsyres.2014.08.019 25219353

[B34] Rosenberg E. C. Patra P. H. Whalley B. J. (2017). Therapeutic effects of cannabinoids in animal models of seizures, epilepsy, epileptogenesis, and epilepsy-related neuroprotection. Epilepsy Behav. 70 (Pt B), 319–327. 10.1016/j.yebeh.2016.11.006 28190698 PMC5651410

[B35] Sachdeo R. C. Glauser T. A. Ritter F. Reife R. Lim P. Pledger G. (1999). A double-blind, randomized trial of topiramate in Lennox-Gastaut syndrome. Topiramate YL Study Group. Neurology 52 (9), 1882–1887. 10.1212/wnl.52.9.1882 10371538

[B36] Sterne J. A. C. Savović J. Page M. J. Elbers R. G. Blencowe N. S. Boutron I. (2019). RoB 2: a revised tool for assessing risk of bias in randomised trials. Bmj 366, l4898. 10.1136/bmj.l4898 31462531

[B37] Thiele E. A. Marsh E. D. French J. A. Mazurkiewicz-Beldzinska M. Benbadis S. R. Joshi C. (2018). Cannabidiol in patients with seizures associated with Lennox-Gastaut syndrome (GWPCARE4): a randomised, double-blind, placebo-controlled phase 3 trial. Lancet 391 (10125), 1085–1096. 10.1016/S0140-6736(18)30136-3 29395273

[B38] Trevathan E. Murphy C. C. Yeargin-Allsopp M. (1997). Prevalence and descriptive epidemiology of Lennox-Gastaut syndrome among Atlanta children. Epilepsia 38 (12), 1283–1288. 10.1111/j.1528-1157.1997.tb00065.x 9578523

[B39] Tupal S. Faingold C. L. (2021). Serotonin 5-HT(4) receptors play a critical role in the action of fenfluramine to block seizure-induced sudden death in a mouse model of SUDEP. Epilepsy Res. 177, 106777. 10.1016/j.eplepsyres.2021.106777 34601387

[B40] US Food and Drug Administration (2018). FDA approves first drug comprised of an active ingredient derived from marijuana to treat rare, severe forms of epilepsy. Available at: https://www.fda.gov/NewsEvents/Newsroom/PressAnnouncements/ucm611046.htm (Accessed October 10, 2018).

[B41] Vázquez M. Maldonado C. Guevara N. Rey A. Fagiolino P. Carozzi A. (2018). Lamotrigine-valproic acid interaction leading to stevens-johnson syndrome. Case Rep. Med. 2018, 5371854. 10.1155/2018/5371854 30228819 PMC6136509

[B42] Zhang H. B. Heckman L. Niday Z. Jo S. Fujita A. Shim J. (2022b). Cannabidiol activates neuronal Kv7 channels. Elife 11, e73246. 10.7554/eLife.73246 35179483 PMC8856652

[B43] Zhang L. Wang J. Wang C. (2022a). Efficacy and safety of antiseizure medication for Lennox-Gastaut syndrome: a systematic review and network meta-analysis. Dev. Med. Child. Neurol. 64 (3), 305–313. 10.1111/dmcn.15072 34590711

