# Supplementary material for: Efficacy and safety of pharmacological and non-pharmacological therapies in Lennox-Gastaut syndrome: a systematic review and network meta-analysis
Source: Front Pharmacol. 2025 Feb 26;16:1522543. doi: 10.3389/fphar.2025.1522543 (PMC11898213; doi:10.3389/fphar.2025.1522543)
Supplement: Supplementary file 1 [file Table1.DOCX]

Table 1 Characteristics of the study participants

| Study | Year | Country | Sample size | Gender(M/F) | Mean age | Intervention | Outcomes |
| --- | --- | --- | --- | --- | --- | --- | --- |
| Glauser | 2008 | USA | Rufinamide:74  UT:64 | 86/52 | Rufinamide:13  UT:10.5 | Rufinamide:45mg/kg/d | F1,F2;F3;F4 |
| Dalic | 2022 | Australia | DBS：10  UT：9 | 6/13 | DBS：24.4  UT：25 | Deep brain stimulation | F1;F2;F3;F4 |
| Liang | 2014 | China | Callosotomy：23  UT：37 | 38/22 | Callosotomy：9.48  UT：9.73 | Anterior corpus callosotomy | F1;F4 |
| Devinsky | 2018 | USA | Cannabidiol:149  UT:76 | 129/96 | Cannabidiol:15.7  UT:15.3 | Cannabidiol:10mg/kg/d  Cannabidiol:20mg/kg/d | F1;F2;F3;F4 |
| Knupp | 2022 | USA | Fenfluramine:176  UT:87 | 146/117 | Fenfluramine:13  UT:14 | Fenfluramine:0.2mg/kg/d  Fenfluramine:0.7mg/kg/d | F1;F2;F3;F4 |
| Felbamate group | 1993 | USA | Felbamate:37  UT:36 | 51/22 | Felbamate:12  UT:14 | Felbamate:45mg/kg/d | F2;F3 |
| Motte | 1997 | USA | Lamotrigine:79  UT:90 | 99/70 | Lamotrigine:9.6  UT:10.9 | Lamotrigine:18mg/kg/d | F2;F3;F4 |
| Ohtsuka | 2016 | Japan | Rufinamide:28  UT:30 | 33/21 | Rufinamide:16  UT:13.9 | Rufinamide:45mg/kg/d | F1;F2;F3;F4 |
| Thiele | 2018 | USA | Cannabidiol：86  UT：85 | 88/83 | Cannabidiol：15.4  UT：15.2 | Cannabidiol:20mg/kg/d | F1;F2;F3;F4 |
| Ng YT | 2011 | USA | Clobazam:179  UT:59 | 144/94 | Clobazam：12.2  UT：13 | Clobazam:0.25mg/kg/d  Clobazam:0.5mg/kg/d  Clobazam:1mg/kg/d | F1;F2;F3;F4 |
| Sachdeo | 1999 | USA | Topiramate：48  UT：50 | 53/45 | Topiramate：11.2  UT：11.2 | Topiramate:6mg/kg/d | F2;F3;F4 |
| McMurray | 2016 | UK | Rufinamide:21  UT:10 | 20/11 | Rufinamide:25.2  UT:29.3 | Rufinamide:45mg/kg/d | F1;F2;F4 |

UT: Usual treatment (sham-operated group or placebo group)

DBS: Deep brain stimulation

F1: Adverse events

F2: Median Percent Reductions in Monthly Drop-Seizure Frequency during the Treatment Period.

F3: Serious adverse events

F4:Reductions of at Least 50% from Baseline in Drop-Seizure Frequency during the Treatment Period.
