# Supplementary figures and images for: Efficacy and safety of pharmacological and non-pharmacological therapies in Lennox-Gastaut syndrome: a systematic review and network meta-analysis
Source: Front Pharmacol. 2025 Feb 26;16:1522543. doi: 10.3389/fphar.2025.1522543 (PMC11898213; doi:10.3389/fphar.2025.1522543)

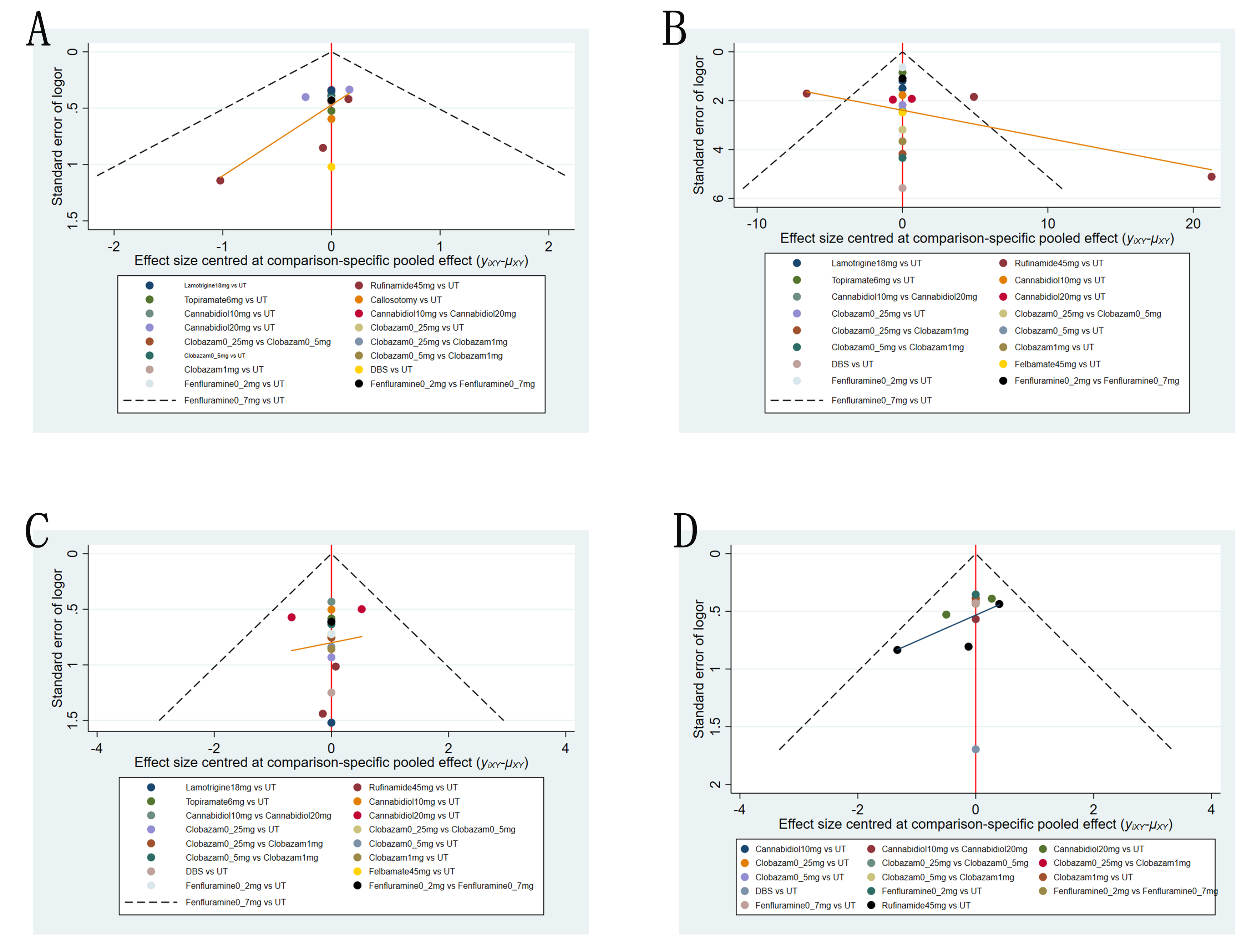

Supplement: Supplementary file 2 [file Image1.TIF]
