# Supplementary material for: Efficacy and safety of pharmacological and non-pharmacological therapies in Lennox-Gastaut syndrome: a systematic review and network meta-analysis
Source: Front Pharmacol. 2025 Feb 26;16:1522543. doi: 10.3389/fphar.2025.1522543 (PMC11898213; doi:10.3389/fphar.2025.1522543)
Supplement: Supplementary file 3 [file Table2.DOCX]

| Treatment | F1(%) | F2(%) | F3(%) | F4(%) |
| --- | --- | --- | --- | --- |
| Cannabidiol:10mg/kg/d | 39.1 | 31.5 | 32.8 | 45.6 |
| Cannabidiol:20mg/kg/d | 7.1 | 47 | 34.5 | 50.9 |
| Clobazam:0.25mg/kg/d | 68.6 | 66.0 | 60.0 | 19.8 |
| Clobazam:0.5mg/kg/d | 23.1 | 85.0 | 35.4 | 51.5 |
| Clobazam:1mg/kg/d | 75.3 | 99.4 | 41.0 | 89.7 |
| DBS | 98.3 | 71.9 | 99.1 | 59.3 |
| Fenfluramine:0.2mg/kg/d | 59.7 | 8.5 | 74.8 | 57.7 |
| Fenfluramine:0.7mg/kg/d | 15.4 | 26.5 | 40.7 | 48.7 |
| Rufinamide:45mg/kg/d | 43.0 | 78.2 | 73.4 | 72.1 |
| Anterior corpus callosotomy | NA | NA | NA | 84.3 |
| Felbamate:45mg/kg/d | NA | 51.8 | 37.3 | NA |
| Lamotrigine:18mg/kg/d | NA | 53.0 | 0.7 | 29.2 |
| Topiramate:6mg/kg/d | NA | 30.6 | 42.1 | 39.0 |
| UT | 70.3 | 0.076 | 78 | 2.1 |
