# Supplementary material for: Efficacy and safety of pharmacological and non-pharmacological therapies in Lennox-Gastaut syndrome: a systematic review and network meta-analysis
Source: Front Pharmacol. 2025 Feb 26;16:1522543. doi: 10.3389/fphar.2025.1522543 (PMC11898213; doi:10.3389/fphar.2025.1522543)
Supplement: Supplementary file 4 [file Table3.docx]

| F1：OR 95%CI |  |  |  |  |  |  |  |  |  |
| --- | --- | --- | --- | --- | --- | --- | --- | --- | --- |
| Cannabidiol10mg |  |  |  |  |  |  |  |  |  |
| 0.46 (0.19, 1.14) | Cannabidiol20mg |  |  |  |  |  |  |  |  |
| 1.77 (0.59, 5.45) | 3.84 (1.44, 10.32)* | Clobazam0_25mg |  |  |  |  |  |  |  |
| 0.71 (0.22, 2.3) | 1.54 (0.53, 4.41) | 0.4 (0.17, 0.93) | Clobazam0_5mg |  |  |  |  |  |  |
| 2.01 (0.67, 6.13) | 4.35 (1.65, 11.67)* | 1.13 (0.53, 2.43) | 2.81 (1.23, 6.76)* | Clobazam1mg |  |  |  |  |  |
| 53054215.79 (1.9, 1.24643653043255e+25)* | 113903619.71 (4.19, 2.64751995632895e+25)* | 29855928.2 (1.08, 6.90652356063824e+24)* | 73458459.28 (2.7, 1.75200741804761e+25)* | 26436248.79 (0.95, 6.1163583862229e+24) | DBS |  |  |  |  |
| 1.48 (0.52, 4.35) | 3.2 (1.27, 8.2)* | 0.84 (0.29, 2.35) | 2.07 (0.7, 6.27) | 0.74 (0.26, 2.05) | 0 (0, 0.77)* | Fenfluramine0_2mg |  |  |  |
| 0.57 (0.17, 1.86) | 1.25 (0.42, 3.5) | 0.32 (0.1, 1.01) | 0.81 (0.24, 2.67) | 0.29 (0.09, 0.88) | 0 (0, 0.3)* | 0.39 (0.16, 0.9)* | Fenfluramine0_7mg |  |  |
| 1.09 (0.39, 3.16) | 2.37 (0.97, 5.89) | 0.62 (0.22, 1.7) | 1.54 (0.53, 4.6) | 0.54 (0.2, 1.48) | 0 (0, 0.56)* | 0.74 (0.28, 1.93) | 1.9 (0.66, 5.78) | Rufinamide45mg |  |
| 1.72 (0.79, 3.94) | 3.74 (2.06, 7.04)* | 0.98 (0.45, 2.1) | 2.43 (1.05, 5.82)* | 0.86 (0.4, 1.84) | 0 (0, 0.87)* | 1.17 (0.58, 2.37) | 2.99 (1.32, 7.38)* | 1.58 (0.82, 3.09) | UT |

| F2: MD 95% CI |  |  |  |  |  |  |  |  |  |  |  |  |
| --- | --- | --- | --- | --- | --- | --- | --- | --- | --- | --- | --- | --- |
| Cannabidiol10mg |  |  |  |  |  |  |  |  |  |  |  |  |
| 1.8 (-2.29, 5.96) | Cannabidiol20mg |  |  |  |  |  |  |  |  |  |  |  |
| 4.62 (-0.94, 10.14) | 2.81 (-2.29, 7.93) | Clobazam0_25mg |  |  |  |  |  |  |  |  |  |  |
| 8.75 (2.81, 14.69)* | 6.94 (1.39, 12.5)* | 4.11 (-2.18, 10.44) | Clobazam0_5mg |  |  |  |  |  |  |  |  |  |
| 18.2 (10.21, 26.21)* | 16.39 (8.73, 24.04)* | 13.58 (5.4, 21.78)* | 9.45 (0.95, 17.94)* | Clobazam1mg |  |  |  |  |  |  |  |  |
| 7.05 (-4.44, 18.49) | 5.22 (-6.08, 16.45) | 2.39 (-9.39, 14.19) | -1.74 (-13.67, 10.19) | -11.18 (-24.26, 1.89) | DBS |  |  |  |  |  |  |  |
| 2.59 (-3.39, 8.55) | 0.79 (-4.79, 6.35) | -2.03 (-8.55, 4.41) | -6.16 (-13.03, 0.68) | -15.61 (-24.31, -6.98)* | -4.46 (-16.41, 7.55) | Felbamate45mg |  |  |  |  |  |  |
| -6.6 (-10.25, -2.92)* | -8.41 (-11.36, -5.45)* | -11.23 (-15.73, -6.71)* | -15.34 (-20.35, -10.32)* | -24.8 (-32.09, -17.48)* | -13.64 (-24.61, -2.59)* | -9.18 (-14.19, -4.15)* | Fenfluramine0_2mg |  |  |  |  |  |
| -0.45 (-4.52, 3.63) | -2.26 (-5.7, 1.19) | -5.08 (-9.88, -0.23)* | -9.2 (-14.49, -3.88)* | -18.64 (-26.14, -11.14)* | -7.48 (-18.58, 3.7) | -3.04 (-8.34, 2.32) | 6.15 (3.8, 8.49)* | Fenfluramine0_7mg |  |  |  |  |
| 2.62 (-1.93, 7.13) | 0.81 (-3.19, 4.78) | -2.01 (-7.25, 3.2) | -6.13 (-11.84, -0.43)* | -15.59 (-23.38, -7.84)* | -4.41 (-15.72, 6.91) | 0.03 (-5.63, 5.69) | 9.22 (5.98, 12.38)* | 3.07 (-0.59, 6.7) | Lamotrigine18mg |  |  |  |
| 6.99 (2.2, 11.18)* | 5.19 (1.03, 8.72)* | 2.34 (-3.19, 7.23) | -1.77 (-7.83, 3.67) | -11.25 (-19.63, -3.66)* | -0.1 (-12.18, 11.16) | 4.39 (-1.7, 9.8) | 13.61 (10.41, 16.18)* | 7.46 (3.7, 10.58)* | 4.37 (-0.02, 8.18) | Rufinamide45mg |  |  |
| 0.05 (-3.76, 3.89) | -1.76 (-4.9, 1.39) | -4.58 (-9.22, 0.08) | -8.7 (-13.82, -3.56)* | -18.15 (-25.53, -10.78)* | -6.99 (-17.99, 4.09) | -2.54 (-7.65, 2.59) | 6.65 (4.54, 8.75)* | 0.49 (-2.23, 3.22) | -2.56 (-5.97, 0.84) | -6.96 (-9.78, -3.51)* | Topiramate6mg |  |
| -9.9 (-13.35, -6.43)* | -11.71 (-14.39, -9.02)* | -14.52 (-18.85, -10.18)* | -18.64 (-23.5, -13.78)* | -28.09 (-35.28, -20.91)* | -16.93 (-27.83, -5.95)* | -12.5 (-17.3, -7.6)* | -3.3 (-4.55, -2.05)* | -9.45 (-11.59, -7.3)* | -12.52 (-15.43, -9.53)* | -16.92 (-19.16, -13.99)* | -9.95 (-11.61, -8.26)* | UT |

| F3: OR 95%CI |  |  |  |  |  |  |  |  |  |  |  |  |
| --- | --- | --- | --- | --- | --- | --- | --- | --- | --- | --- | --- | --- |
| Cannabidiol10mg |  |  |  |  |  |  |  |  |  |  |  |  |
| 1.06 (0.47, 2.34) | Cannabidiol20mg |  |  |  |  |  |  |  |  |  |  |  |
| 2.21 (0.22, 19.47) | 2.1 (0.22, 16.76) | Clobazam0_25mg |  |  |  |  |  |  |  |  |  |  |
| 1.05 (0.11, 6.76) | 1 (0.12, 5.8) | 0.48 (0.09, 1.99) | Clobazam0_5mg |  |  |  |  |  |  |  |  |  |
| 1.24 (0.13, 8.38) | 1.18 (0.13, 7.26) | 0.56 (0.1, 2.52) | 1.17 (0.33, 4.41) | Clobazam1mg |  |  |  |  |  |  |  |  |
| 60.37 (4.62, 2151.06)* | 56.93 (4.78, 1951.22)* | 28.26 (1.25, 1576.9)* | 59.96 (3.13, 3195.91)* | 51.19 (2.61, 2681.81)* | DBS |  |  |  |  |  |  |  |
| 1.1 (0.17, 5.96) | 1.04 (0.17, 5.05) | 0.49 (0.04, 6.37) | 1.04 (0.11, 12.08) | 0.89 (0.09, 10.48) | 0.02 (0, 0.29)* | Felbamate45mg |  |  |  |  |  |  |
| 3.76 (0.63, 22.55) | 3.56 (0.67, 19.52) | 1.7 (0.15, 22.83) | 3.6 (0.4, 43.85) | 3.06 (0.32, 37.72) | 0.06 (0, 1.04) | 3.44 (0.44, 31.42) | Fenfluramine0_2mg |  |  |  |  |  |
| 1.28 (0.25, 5.82) | 1.21 (0.26, 4.93) | 0.57 (0.05, 6.43) | 1.21 (0.15, 12.41) | 1.03 (0.12, 10.82) | 0.02 (0, 0.3)* | 1.16 (0.17, 8.73) | 0.34 (0.09, 1.1) | Fenfluramine0_7mg |  |  |  |  |
| 0 (0, 0.38)* | 0 (0, 0.36)* | 0 (0, 0.2)* | 0 (0, 0.41)* | 0 (0, 0.36)* | 0 (0, 0.01)* | 0 (0, 0.37)* | 0 (0, 0.11)* | 0 (0, 0.3)* | Lamotrigine18mg |  |  |  |
| 3.92 (0.51, 29.07) | 3.71 (0.53, 25.4) | 1.76 (0.13, 27.62) | 3.75 (0.34, 53.88) | 3.19 (0.27, 46.8) | 0.06 (0, 1.27) | 3.59 (0.36, 38.92) | 1.03 (0.1, 10.69) | 3.08 (0.36, 28.3) | 5686802661.03 (9.35, 1.57734613767195e+31)* | Rufinamide45mg |  |  |
| 1.3 (0.27, 5.74) | 1.23 (0.29, 4.78) | 0.58 (0.06, 6.52) | 1.23 (0.16, 12.59) | 1.05 (0.13, 10.91) | 0.02 (0, 0.31)* | 1.19 (0.18, 8.66) | 0.34 (0.05, 2.29) | 1.02 (0.18, 5.98) | 1881784518.59 (3.37, 4.9354571449587e+30)* | 0.33 (0.04, 2.8) | Topiramate6mg |  |
| 3.65 (1.43, 9.54)* | 3.44 (1.7, 7.39)* | 1.64 (0.24, 13.91) | 3.42 (0.71, 26.49) | 2.92 (0.56, 22.95) | 0.06 (0, 0.65)* | 3.29 (0.83, 17.22) | 0.97 (0.21, 4.41) | 2.83 (0.89, 11.13) | 5328621584.77 (10.07, 1.43808954714402e+31)* | 0.93 (0.16, 5.66) | 2.8 (0.91, 9.82) | UT |

| F4: OR 95% CI |  |  |  |  |  |  |  |  |  |  |  |  |
| --- | --- | --- | --- | --- | --- | --- | --- | --- | --- | --- | --- | --- |
| Callosotomy |  |  |  |  |  |  |  |  |  |  |  |  |
| 2.52 (0.65, 10.63) | Cannabidiol10mg |  |  |  |  |  |  |  |  |  |  |  |
| 2.31 (0.66, 9.09) | 0.92 (0.48, 1.73) | Cannabidiol20mg |  |  |  |  |  |  |  |  |  |  |
| 4.27 (1.06, 18.8)* | 1.69 (0.59, 4.8) | 1.85 (0.72, 4.7) | Clobazam0_25mg |  |  |  |  |  |  |  |  |  |
| 2.29 (0.57, 9.98) | 0.91 (0.32, 2.55) | 0.99 (0.39, 2.48) | 0.54 (0.25, 1.14) | Clobazam0_5mg |  |  |  |  |  |  |  |  |
| 0.92 (0.21, 4.21) | 0.36 (0.12, 1.09) | 0.4 (0.14, 1.07) | 0.22 (0.09, 0.5)* | 0.4 (0.16, 0.93)* | Clobazam1mg |  |  |  |  |  |  |  |
| 1.76 (0.13, 18.92) | 0.7 (0.06, 5.95) | 0.76 (0.07, 6.11) | 0.41 (0.03, 3.62) | 0.77 (0.06, 6.75) | 1.93 (0.15, 17.63) | DBS |  |  |  |  |  |  |
| 2.05 (0.48, 9.31) | 0.81 (0.26, 2.38) | 0.89 (0.32, 2.33) | 0.48 (0.15, 1.5) | 0.9 (0.28, 2.77) | 2.23 (0.65, 7.6) | 1.16 (0.13, 14.08) | Fenfluramine0_2mg |  |  |  |  |  |
| 2.37 (0.55, 10.79) | 0.94 (0.3, 2.78) | 1.03 (0.37, 2.73) | 0.56 (0.17, 1.76) | 1.04 (0.32, 3.26) | 2.59 (0.75, 8.89) | 1.34 (0.15, 16.48) | 1.16 (0.59, 2.28) | Fenfluramine0_7mg |  |  |  |  |
| 3.44 (0.89, 14.46) | 1.36 (0.52, 3.63) | 1.49 (0.63, 3.5) | 0.81 (0.28, 2.28) | 1.5 (0.53, 4.24) | 3.76 (1.24, 11.75)* | 1.95 (0.23, 23.05) | 1.68 (0.57, 5.21) | 1.45 (0.49, 4.49) | Lamotrigine18mg |  |  |  |
| 1.54 (0.4, 6.52) | 0.61 (0.23, 1.62) | 0.67 (0.28, 1.56) | 0.36 (0.13, 1.03) | 0.67 (0.24, 1.91) | 1.68 (0.55, 5.27) | 0.87 (0.1, 10.31) | 0.75 (0.25, 2.31) | 0.65 (0.22, 2) | 0.45 (0.17, 1.19) | Rufinamide45mg |  |  |
| 2.93 (0.61, 14.7) | 1.17 (0.32, 3.99) | 1.27 (0.39, 3.97) | 0.69 (0.18, 2.5) | 1.28 (0.34, 4.65) | 3.2 (0.81, 12.66) | 1.67 (0.17, 22.02) | 1.44 (0.37, 5.51) | 1.24 (0.32, 4.77) | 0.85 (0.24, 2.94) | 1.9 (0.53, 6.68) | Topiramate6mg |  |
| 7.11 (2.28, 25.45)* | 2.83 (1.43, 5.69)* | 3.09 (1.87, 5.17)* | 1.68 (0.77, 3.68) | 3.12 (1.46, 6.83)* | 7.78 (3.33, 19.54)* | 4.04 (0.54, 43.54) | 3.47 (1.54, 8.54)* | 3 (1.32, 7.39)* | 2.07 (1.05, 4.18)* | 4.61 (2.35, 9.61)* | 2.43 (0.88, 7.2) | UT |
